# The Modified Risk of Paradoxical Embolism Score Is Associated with Patent Foramen Ovale in Patients with Ischemic Stroke: A Nationwide US Analysis

**DOI:** 10.3390/jcdd11070213

**Published:** 2024-07-05

**Authors:** Chun Shing Kwok, Zaheer Alisiddiq, Maximilian Will, Konstantin Schwarz, Chee Khoo, Adrian Large, Robert Butler, Gregory Y. H. Lip, Adnan I. Qureshi, Josip Andelo Borovac

**Affiliations:** 1 Department of Cardiology, University Hospitals of North Midlands NHS Foundation Trust, Stoke-on-Trent ST4 6QG, UK; shingkwok@doctors.org.uk (C.S.K.); zaheeralisiddiq@gmail.com (Z.A.); chee.khoo@uhnm.nhs.uk (C.K.); adrian.large@uhnm.nhs.uk (A.L.); rob.butler@uhnm.nhs.uk (R.B.); 2 Department of Internal Medicine 3, University Hospital St. Pölten, Karl Landsteiner University of Health Sciences, 3100 Krems, Austria; maximilian.will@me.com (M.W.); konstantin.schwarz@gmx.net (K.S.); 3 Liverpool Centre for Cardiovascular Science, University of Liverpool, Liverpool John Moores University and Liverpool Heart & Chest Hospital, Liverpool L69 3BX, UK; gregory.lip@gliverpool.ac.uk; 4 Danish Center for Health Services Research, Department of Clinical Medicine, Aalborg University, 9220 Aalborg, Denmark; 5 Zeenat Qureshi Stroke Institute and Department of Neurology, University of Missouri, Columbia, MO 65211, USA; qureshai@gmail.com; 6 Division of Interventional Cardiology, Cardiovascular Diseases Department, University Hospital of Split, 21000 Split, Croatia

**Keywords:** acute ischemic stroke, AIS, cryptogenic stroke, patent foramen ovale, PFO, RoPE score, risk of paradoxical embolism, risk stratification, prediction

## Abstract

Background: The Risk of Paradoxical Embolism (RoPE) score was developed to identify stroke-related patent foramen ovale (PFO) in patients with cryptogenic stroke. Methods: We conducted a retrospective analysis of the 2016 to 2020 National Inpatient Sample to determine the performance of the modified RoPE score in identifying the presence of a PFO in patients with acute ischemic stroke (AIS). Results: A total of 3,338,805 hospital admissions for AIS were analysed and 3.0% had PFO. Patients with PFO were younger compared to those without a PFO (median 63 years vs. 71 years, *p* < 0.001) and fewer were female (46.1% vs. 49.7%, *p* < 0.001). The patients with PFO had greater mean modified RoPE scores (4.0 vs. 3.3, *p* < 0.001). The area under the curve for the RoPE score in predicting PFOs was 0.625 (95%CI 0.620–0.629). The best diagnostic power of the RoPE score was achieved with a cut-off point of ≥4 where the sensitivity was 55% and the specificity was 64.2%. A cut-off point of ≥5 increased the specificity (83.1%) at the expense of sensitivity (35.8%). The strongest predictor of PFOs was deep vein thrombosis (OR 3.97, 95%CI 3.76–4.20). Conclusions: The modified RoPE score had modest predictive value in identifying patients with PFO among patients admitted with AIS.

## 1. Introduction

Patent foramen ovale (PFO) is the most common congenital heart abnormality of foetal origin and it is present in approximately 25% of the worldwide adult population [1]. It holds clinical relevance since a PFO is a highly prevalent finding in cryptogenic ischemic stroke, particularly among younger adults [2]. A classical cardioembolic presentation due to PFO includes the onset of symptoms after a Valsalva-provoking activity such as coughing, bending or similar which corresponds to paradoxical embolism facilitated by the transient rise in the right atrial pressure and the co-occurrence of cerebral and systemic emboli [3]. A case-control study of 60 adults under 55 years of age with ischemic stroke matched to a control group of 100 patients found that a PFO was present in 40% of patients with ischemic stroke compared to 10% of controls [4]. A subsequent prospective evaluation of 227 patients with cryptogenic stroke and 276 control patients with a stroke of known cause found that the odds of having a PFO were significantly greater for patients with cryptogenic stroke for both younger patients (OR 4.70, 95%CI 1.89–11.68) and older patients (OR 2.92, 95%CI 1.70–5.01) [5]. A meta-analysis of 15 studies revealed that PFO presence was associated with an increase in the odds of experiencing an ischemic stroke compared to controls (OR 1.83, 95%CI 1.25–2.66) [6]. There is, therefore, significant interest in predicting PFO among patients with ischemic stroke since there is growing evidence that PFO closure may reduce the risk of recurrent stroke in carefully selected young cryptogenic stroke patients [7].

The Risk of Paradoxical Embolism (RoPE) score was derived from 12 component studies to determine the likelihood that a stroke was related to a PFO [8]. The score consists of six variables with a maximum score of 10 points. In the derivation study it was found that among patients with 0 to 3 points, a PFO existed in 23% of patients to 73% prevalence of PFO in patients with scores of 9 or 10 [8]. In the validation study, using a cut-off of 7 points with area under the curve of 0.704, there was a sensitivity of 69.4% and specificity of 62.5%, and a RoPE score >7 was significantly associated with greater PFO frequency (53.6% vs. 10%) [9]. Using data from three clinical trials of PFO closure, the RoPE score identified patients with cryptogenic stroke who are likely to have a PFO that is pathogenic rather than incidental [10].

Whether or not the RoPE score can be used to predict patients with a PFO among patients with ischemic stroke from a large and real-world nationwide patient sample is unknown and has not been yet explored. For these reasons, in the present study, we examined the predictive value of a modified RoPE score in identifying the presence of a PFO in patients with ischemic stroke from the large and representative nationwide database in the United States—the National Inpatient Sample (NIS).

## 2. Materials and Methods

This manuscript was prepared in accordance with the recommendations of the STROBE criteria [11].

### 2.1. Ethics and Approvals

Institutional review board approval for the study was not required for analysis of data from that National Inpatient Sample [12].

### 2.2. Dataset

We analysed data from the NIS between 2016 to 2020. The NIS is the largest all-payer inpatient care database in the United States which is produced by the Healthcare Cost and Utilization Project (HCUP) at the Agency for Healthcare Research and Quality (AHRQ). The NIS samples hospital admission data from approximately 20% of the hospitals in the United States which translates to 5 to 8 million hospital admissions from approximately 1000 hospitals. It contains more than 100 clinical and non-clinical variables including diagnostic codes, procedure codes, patient demographics and patient admission and discharge statuses. The data from the NIS can be weighted to generate national estimates.

### 2.3. Variable Definition

We used International Classification of Diseases, 10th Revision, Clinical Modification (ICD-10-CM) primary diagnosis code I63 to identify hospital admissions where patients were diagnosed with acute ischemic stroke. The modified RoPE score was defined by a history of hypertension, history of diabetes, history of stroke or TIA, smoker and age excluding cortical infarct on imaging. Each variable aside from age was assigned one point for yes and age was scored up to 5 points (18–29 years 5 points, 30–39 years 4 points, 40–49 years 3 points, 50–59 years 2 points, 60–69 years 1 point and ≥70 years 0 points). The exclusion of the cortical infarct on imaging was applied because our cohort was patients with hospital admission for acute ischemic stroke which likely had cortical infarction on imaging. The modified RoPE score was scored from 0 to a maximum score of 9.

Clinical comorbidity variables for each admission were determined based on the ICD-10 codes or data available in the NIS as defined in Table A1 (Appendix A). We identified the following neurological deficits using ICD-10-diagnosis codes as indicators of neurological severity: aphasia (I69.320, I69.920, R47.01), hemiplegia (I69.35, G81), neglect (R41.4), somnolence, stupor and coma (R40), dysphagia (R13.1) and homonymous hemianopsia (H53.46). We also evaluated the proportion of patients admitted to the teaching and non-teaching hospitals, and those admitted to rural hospitals. The hospitals’ locations and teaching statuses in the NIS database were obtained from the American Hospital Association Annual Survey of Hospitals. A metropolitan statistical area is considered to be urban, and a non-metropolitan statistical area is rural. Teaching hospitals have an AMA-approved residency program or have membership in the council of teaching hospitals. The hospital bed sizes were classified in the NIS database into three subgroups (small, medium, or large) based on the location and teaching status of the hospital. Patients undergoing interhospital transfer were identified using the “TRAN_IN” variable. Intracranial haemorrhage was defined as a composite of intracerebral or subarachnoid haemorrhage as identified using ICD-10-CM codes I60, I61 and I62.9.

The main outcome measure for this analysis was the presence of PFO defined by the ICD-10 diagnosis code Q21.1. We also assessed whether patients were discharged home or died in hospital based on the “DISPUNIFORM” variable. A full description of all the variables and codes is shown in Table A1 (Appendix A).

### 2.4. Statistical Analysis

Statistical analysis was performed on STATA 13.0 (College Station, TX, USA). Descriptive statistics were presented for the patient characteristics, management and outcomes stratified by the presence or absence of PFO. For continuous variables the median and interquartile range were presented and the median test on Stata was used to determine if there were statistical differences between the groups with and without PFO. Categorical variables were described with percentages and the Chi2 test was used for determining if there were differences for the group with PFO. The modified RoPE scores for the cohort according to presence or absence of PFO are shown graphically and in a table. The Stata function “ROCTAB” was used to perform nonparametric ROC analysis which enabled evaluation of the sensitivity and specificity at different cut-off points of modified RoPE score and graphical presentation of the ROC and the area under the curve (AUC). Multiple logistic regression was performed to identify the independent predictors of PFO with patient demographic, hospital, comorbidities, and severity markers as candidate variables in the multivariable-adjusted model.

## 3. Results

A total of 3,351,630 patients with hospital admissions for the principal diagnosis of acute ischemic stroke were identified in the National Inpatient Sample between 2016 to 2020. After excluding 12,825 patients who less than 18 years of age, a total of 3,338,805 hospital admission for acute ischemic stroke were included in the analysis, of which 101,290 (3.0%) had a PFO.

The patient characteristics, management and outcomes for the patients with admission for acute ischemic stroke according to the presence of a PFO are shown in Table 1. Patients with a PFO were younger compared to those without a PFO and fewer were female. A lesser proportion of those with a PFO were receiving Medicare and a greater proportion had private insurance or were self-paying for their treatment. A greater proportion of patients with a PFO were admitted to hospitals with a large bed size and to teaching hospitals. Patients with a PFO were less likely to have hypertension, diabetes mellitus, congestive heart failure, chronic kidney disease and dementia. Similarly, patients with a PFO had a greater prevalence of DVT compared to patients without a PFO. In terms of hospital management, patients with a PFO were more likely to have hospital transfer, thrombolysis, mechanical thrombectomy, and PFO closure. In addition, a greater proportion of patients with a PFO were discharged home, and less likely to die in hospital.

### 3.1. RoPE Score

**Figure 1:**
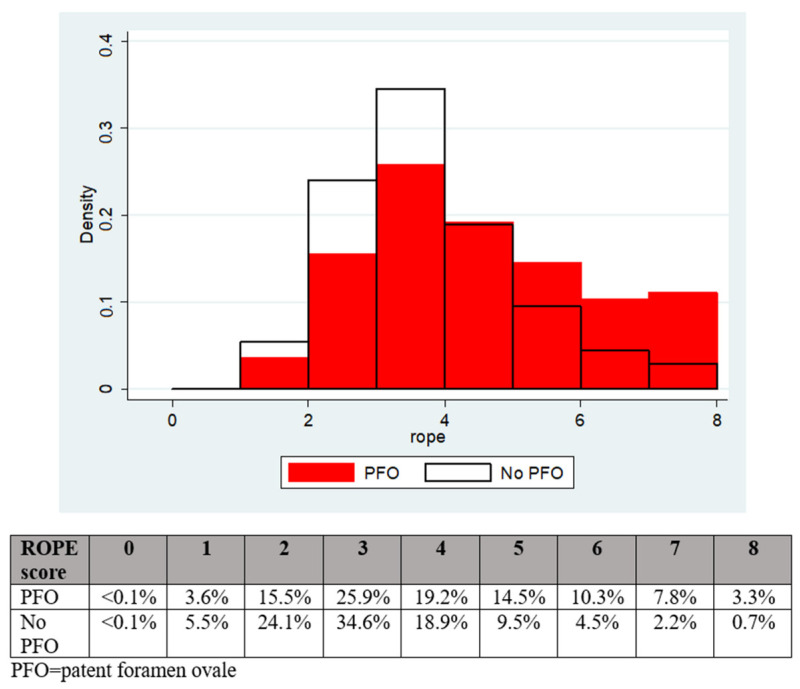
The distribution of RoPE scores according to PFO presence or absence.

The patients with a PFO had a significantly greater mean modified RoPE score (4.0 vs. 3.3, *p* < 0.001) and median modified RoPE score (4 vs. 3, *p* < 0.001) compared to patients without a PFO, as shown in Figure 1.

**Figure 2:**
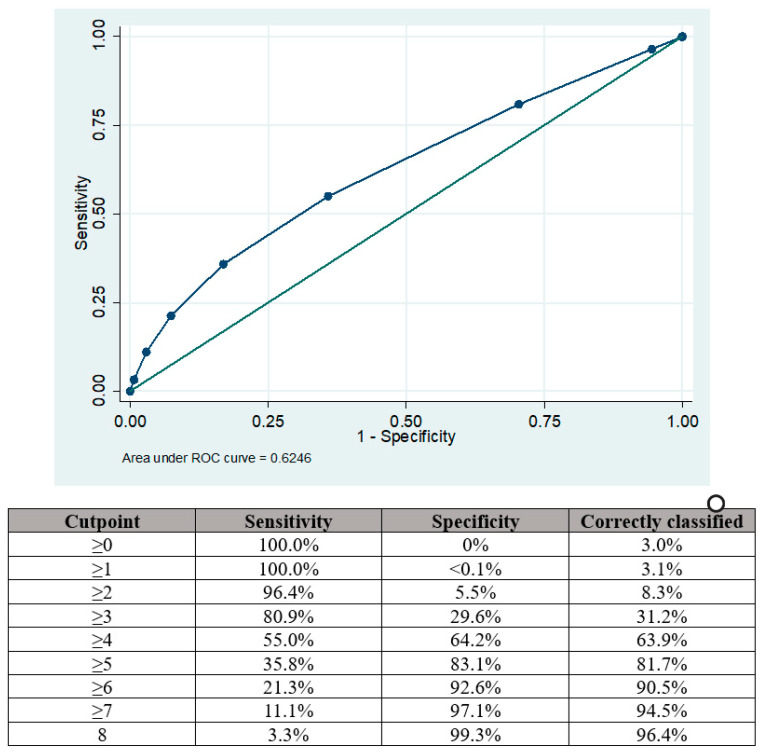
Area under the curve (AUC) analysis showing sensitivities and specificities for PFO presence according to the points aggregated by the RoPE score.

The area under the curve (AUC) for the modified RoPE score in predicting the presence of a PFO among patients with acute ischemic stroke was 0.625 95%CI 0.620–0.629, as depicted in Figure 2.

The optimal balance of sensitivity was achieved with a cut-off point of ≥4 when the sensitivity and specificity for PFO presence were 55.0% and 64.2%, respectively. Sensitivity could be improved by using a cut-off point of ≥3 (80.9%) but there was a trade-off reduction in specificity (29.6%). Similarly, using a cut-off point of ≥5 increased specificity (83.1%) at the expense of sensitivity (35.8%).

### 3.2. Multivariable Regression Analysis

Variables independently associated with PFO presence that were derived from multivariable-adjusted logistic regression analyses are shown in Table 2.

The strongest independent predictors of PFO presence were deep vein thrombosis (OR 3.97, 95%CI 3.76–4.20, *p* < 0.001), atherosclerosis of the aorta (OR 2.35, 95%CI 2.14–2.58, *p* < 0.001), teaching hospital status (OR 1.50, 95%CI 1.44–1.56, *p* < 0.001) and homonymous hemianopsia (OR 1.43, 95%CI 1.33–1.54, *p* < 0.001).

Several variables were associated with a reduction in the odds of having a PFO including age (OR 0.97, 95%CI 0.97–0.97, *p* < 0.001), arterial hypertension (OR 0.71, 95%CI 0.68–0.73, *p* < 0.001), diabetes mellitus (OR 0.71, 95%CI 0.69–0.74, *p* < 0.001) and palliative care (OR 0.57, 95%CI 0.53–0.61, *p* < 0.001).

## 4. Discussion

This large nationwide and real-world observational analysis provides several key findings. First, in the national setting in the United States, 3% of patients with ischemic stroke are found to have a PFO. Second, the patients with ischemic stroke who have a PFO are different from those without a PFO as they are younger and less comorbid. Third, the modified ROPE score provided only a modest predictive value for PFOs among ischemic stroke patients. Finally, the strongest predictor of PFOs in patients with stroke was DVT which was associated with a four-fold increase in the odds of having a PFO. These findings suggest that the modified ROPE score is predictive of a PFO in patients with acute ischemic stroke but not as highly predictive as in some previous validation studies.

The RoPE score places great emphasis on age as well as other factors which are not known to be associated with PFO. A review suggest that the main risk factors linked with PFO-attributed strokes are young age, PFO size, right-to-left shunt degree, PFO morphology, presence of atrial septal aneurysm, intrinsic coagulation-anticoagulation systems imbalance and co-existence of other atrial abnormalities, such as right atrial septal pouch, Eustachian valve and Chiari’s network [13]. Among the non-age factors in the RoPE score, previous stroke or TIA and smoking merit discussion. A meta-analysis of 14 studies suggests that medically treated patients with a PFO do not have a higher risk for recurrent cryptogenic cerebrovascular events, compared with those without a PFO [14].

However, the fact that is a recurrent stroke risk raises the question of whether treatment of the PFO could have prevented the subsequent stroke. There is evidence that tobacco smoking induces a systemic hypercoagulable state [15]. However, the smoking variable in the models is simply treated in a binary manner, and does not consider differences between current smokers and ex-smokers nor the duration of smoking and amount that was smoked (pack–years). Nevertheless, the RoPE score should be considered in the choice of therapy for PFO treatment according to a European positional paper for the management of PFOs [16].

The finding that DVT is the strongest predictor of PFOs in the ischemic stroke cohort is not surprising. DVT in the leg might embolize from the venous circulation and cross via the PFO into the arterial circulation causing an embolic ischemic stroke. However, patients with stroke may also have hemiplegia which may increase their risk of developing DVT. A systematic review suggests that the incidence of DVT varies from 2% to 10% after an acute stroke and death from pulmonary embolism can account for 13% to 2% of early deaths [17]. In the current dataset, we know that both pathologies occurred, but we do not know the temporal sequence of either pathology. This can be challenging to determine as one study suggests that up 17.6% of patients with acute stroke have DVT on admission for an acute stroke [18]. Also, the aetiology of DVT involves Virchow’s triad of stasis, hypercoagulability and endothelial injury, which may also act as precipitating factors of an acute ischemic stroke [19]. Even if a clinical DVT manifests after a stroke it could have started before the onset of stroke. In addition, it is unknown whether patients who have a stroke as a result of paradoxical embolism are at an increased risk of venous thromboembolism complicating their stroke. If there is such an increased risk of venous thromboembolism complicating a stroke related to paradoxical embolism, there may be a benefit to using anticoagulation rather than antiplatelet medications in these patients but this needs to be evaluated in future studies.

An unexpected observation was the association between atherosclerosis of the aorta and PFO. It is unclear why there is this association and the possible mechanism so more studies are needed.

It should be noted that a patient with a PFO is usually clinically silent and clinically asymptomatic but, on the other hand, may cause a paradoxical embolism and is a risk factor mainly for non-lacunar cryptogenic cerebral ischemia in young adults [20]. From a clinical perspective, the finding of a PFO is often an incidental finding, as it is common but the question of whether anything should be done about a PFO once found is clinically relevant. If a patient would not have a change in management if a PFO was found, the patient should not undergo testing for it. The closure of the PFO is now an established percutaneous procedure but there should be strong evidence that undertaking the procedure will benefit the patient because the procedure carries procedural and periprocedural risks. The Society of Cardiovascular Angiography and Intervention (SCAI) guidelines suggest that PFO closure should be preferred over medical therapy alone in persons with systemic embolism without a prior PFO-associated stroke in whom other embolic aetiology have been excluded and in patients between the ages of 18 and 60 years as well as those over age of 60 years with prior PFO-associated stroke [21]. In addition, these guidelines suggests that a RoPE score of ≥7 may identify patients who are likely to receive a greater benefit from PFO closure. The European positional paper states that percutaneous closure of PFO can be performed in carefully selected patients aged 18 to 65 years with a confirmed cryptogenic stroke, systemic embolism and an estimated high probability of a causal role of the PFO as assessed by clinical, anatomical and imaging features [16]. This statement also mentions the RoPE score as a tool which may be useful but there are no large external validation studies supporting its use.

In the present study, we utilized the National Inpatient Sample data in an attempt to validate the modified RoPE score and we found that it does not perform as well as previous validation studies for predicting PFOs in cryptogenic stroke patients. One possible reason for this may be that this cohort lacks the granular detail to determine what proportion of the strokes did not have an identifiable cause and the inclusion of those with identifiable precipitants for stroke may explain its modest predictive results for PFO. Selection biases in real-world testing for PFO may explain some of the findings of the current study. We found that younger patients and those with private insurance were more likely to have a PFO. This may reflect the greater use of testing for PFOs among younger patients and those who have their care paid for by insurance. The lower proportion of patients with dementia in the PFO group may reflect the younger population with PFOs. It is notable that there may be undertesting of PFOs in the study as the overall rate of PFOs of 3% is low, but the cohort is not exclusively patients with cryptogenic stroke.

### Strengths and Limitations

This study has several strengths and limitations. The strength of the current evaluation is that is represents a large contemporary real-world ischemic stroke population that has nationally representative data from the United States. Also, we were able to evaluate many different variables which are clinically relevant for patients with acute ischemic stroke. The first limitation is that we do not have imaging data, so we had to modify the original RoPE score as one of the variables was cortical infarcts on imaging. However, we suspect that the vast majority of the patients included had cortical infarcts on imaging as they all had a discharge diagnosis of acute ischemic stroke. The second key limitation is that the extent to which PFOs were investigated in the cohort is not known. For patients to have a diagnosis of a PFO on their discharge summary together with acute ischemic stroke, it is likely that the diagnosis was based on an imaging procedure as opposed to a clinical diagnosis. However, what is unclear is how often imaging was undertaken to look for PFOs when more common causes for stroke were identified such as atrial fibrillation, carotid dissection or carotid, cerebral, vertebral, or basilar artery stenoses. Moreover, there may be biases in the investigations into PFOs because only those who may be candidates for PFO closure might undergo testing. As a result, this study may under capture the true number of cases of PFO in the cohort as not all patients may have been tested. Similarly, the use of ICD-10 codes from discharge summaries may be limited because not all patients undergo evaluation for stroke, the components of the ROPE score and PFO. Finally, we need to acknowledge the retrospective and observational nature of the study that may be affected by confounding. We included a variety of potentially relevant variables but there may be unmeasured confounders which affect the results.

## 5. Conclusions

In this nationally representative data from the United States, we found that the modified RoPE score had a modest predictive value in identifying PFO presence among patients admitted with an ischemic stroke. The strongest predictor of PFOs in real-world settings of acute stroke patients was DVT. Future studies are needed to determine how valuable the RoPE score may be in the selection of patients who may benefit from investigations for PFO and PFO closure from real-world settings. In this setting, large prospective observational studies and nationwide registries might be the useful way to go with respect to investigating the occurrence of PFOs among patients who suffered stroke. In the future, such information might help in aiding risk stratification strategies and therapeutic management in terms of percutaneous PFO closure.

## Figures and Tables

**Table 1:** Characteristics, management and outcomes for patients with acute ischemic stroke according to the presence or absence of a patent foramen ovale (PFO).

Variable	No PFO (n = 3,237,515)	PFO (n = 101,290)	*p*-Value
Median age [IQR]	71 [61 to 81]	63 [52 to 74]	<0.001
Female	49.7%	46.1%	<0.001
Race			<0.001
White	67.8%	70.8%	
Black	17.5%	15.3%	
Hispanic	8.4%	8.6%	
Asian or Pacific Islander	3.1%	2.3%	
Native American	0.5%	0.6%	
Other	2.7%	2.5%	
Elective admission	4.7%	4.1%	<0.001
Weekend admission	25.4%	25.5%	0.850
Season			<0.001
Spring	24.9%	23.8%	
Summer	24.9%	25.9%	
Fall	24.9%	25.6%	
Winter	25.3%	24.8%	
Primary expected payer			<0.001
Medicare	65.4%	48.3%	
Medicaid	9.8%	12.3%	
Private insurance	18.3%	31.5%	
Self-pay	3.9%	4.6%	
No charge	0.3%	0.4%	
Other	2.4%	2.9%	
ZIP income quartile			<0.001
1st to 25th	31.2%	27.6%	
26th to 50th	26.5%	25.5%	
51st to 75th	23.5%	25.3%	
76th to 100th	18.8%	21.6%	
Hospital region			<0.001
Northeast	17.3%	19.2%	
Midwest	21.4%	25.8%	
South	42.4%	37.3%	
West	18.9%	17.8%	
Hospital bed size			<0.001
Small	17.3%	13.5%	
Medium	28.8%	26.4%	
Large	53.9%	60.1%	
Rural hospital	17.4%	15.9%	<0.001
Teaching hospital	72.8%	81.6%	<0.001
Nicotine dependence	1.1%	1.1%	0.75
Alcohol misuse	2.5%	2.9%	<0.001
Obesity	13.6%	14.1%	0.035
Hypertension	84.3%	72.6%	<0.001
Hypercholesterolaemia	57.0%	55.0%	<0.001
Diabetes mellitus	39.4%	29.3%	<0.001
Previous myocardial infarction	7.4%	5.6%	<0.001
Congestive heart failure	19.9%	13.4%	<0.001
Atrial fibrillation	26.7%	16.2%	<0.001
Previous stroke	25.0%	25.2%	0.64
Atherosclerosis of the aorta	1.5%	2.7%	<0.001
Peripheral vascular disease	4.5%	3.2%	<0.001
Chronic kidney disease	20.4%	13.8%	<0.001
Liver failure	1.1%	1.0%	0.030
Chronic lung disease	17.2%	16.2%	<0.001
Cancer	6.5%	6.7%	0.224
Dementia	12.8%	6.5%	<0.001
Aphasia	21.4%	24.3%	<0.001
Hemiplegia	45.0%	43.7%	<0.001
Neglect	2.2%	2.8%	<0.001
Stupor	13.1%	13.7%	0.021
Dysphagia	14.0%	12.6%	<0.001
Homonymous hemianopsia	2.5%	4.11%	<0.001
Deep vein thrombosis	2.6%	9.7%	<0.001
Urinary tract infection	12.1%	8.4%	<0.001
Pneumonia	6.4%	5.1%	<0.001
Palliative care	8.9%	4.2%	<0.001
Intubation	6.5%	4.7%	<0.001
Tracheostomy	0.8%	0.7%	0.263
Hospital transfer	16.5%	19.9%	<0.001
Thrombolysis	7.8%	10.2%	<0.001
Mechanical thrombectomy	4.5%	5.9%	<0.001
PFO closure	0%	1.7%	<0.001
Intracranial haemorrhage	5.8%	6.2%	0.012
Discharge home self-care	31.5%	45.2%	<0.001
In-hospital mortality	6.8%	3.1%	<0.001
Mean modified ROPE	3.3 ± 1.4	4.0 ± 1.7	<0.001
Median modified ROPE [IQR]	3 [2 to 4]	4 [3 to 5]	<0.001

Abbreviations: IQR = interquartile range.

**Table 2:** Multivariable predictors of patent foramen ovale (PFO) presence.

Variable	Odds Ratio (95%CI)	*p*-Value
Age (years)	0.97 (0.97–0.97)	<0.001
Race vs. White		
Black	0.75 (0.72–0.78)	<0.001
Asian or Pacific Islander	0.69 (0.62–0.76)	<0.001
Other	0.77 (0.70–0.85)	<0.001
Elective admission	0.86 (0.80–0.93)	<0.001
Season vs. Spring		
Summer	1.07 (1.03–1.12)	0.001
Fall	1.07 (1.03–1.12)	0.001
Winter	1.04 (1.00–1.09)	0.049
Primary expected payer vs. Medicare		
Medicaid	0.86 (0.81–0.91)	<0.001
Private insurance	1.19 (1.15–1.25)	<0.001
Self-pay	0.87 (0.81–0.94)	0.001
ZIP income quartile vs. 1st to 25th		
26th to 50th	1.11 (1.06–1.16)	<0.001
51st to 75th	0.86 (0.83–0.90)	<0.001
76th to 100th	0.83 (0.79–0.87)	<0.001
Hospital region vs. Northeast		
Midwest	1.11 (1.06–1.16)	<0.001
South	0.86 (0.83–0.90)	<0.001
West	0.83 (0.79–0.87)	<0.001
Hospital bed size vs. Small		
Medium	1.20 (1.14–1.26)	<0.001
Large	1.37 (1.31–1.43)	<0.001
Teaching hospital	1.50 (1.44–1.56) *	<0.001
Nicotine dependence	0.81 (0.71–0.94)	0.004
Alcohol misuse	0.88 (0.81–0.96)	0.004
Obesity	0.90 (0.87–0.94)	<0.001
Hypertension	0.71 (0.68–0.73)	<0.001
Hypercholesterolaemia	1.16 (1.13–1.20)	<0.001
Diabetes mellitus	0.71 (0.69–0.74)	<0.001
Previous myocardial infarction	0.86 (0.81–0.92)	<0.001
Congestive heart failure	0.85 (0.82–0.89)	<0.001
Atrial fibrillation	0.74 (0.71–0.77)	<0.001
Previous stroke	1.20 (1.16–1.24)	<0.001
Atherosclerosis of the aorta	2.35 (2.14–2.58) *	<0.001
Peripheral vascular disease	0.80 (0.74–0.87)	<0.001
Chronic kidney disease	0.92 (0.88–0.96)	<0.001
Liver failure	0.78 (0.67–0.91)	0.001
Chronic lung disease	1.07 (1.03–1.12)	0.001
Cancer	0.82 (0.77–0.88)	<0.001
Dementia	0.79 (0.75–0.84)	<0.001
Aphasia	1.24 (1.19–1.28)	<0.001
Hemiplegia	0.93 (0.90–0.96)	<0.001
Neglect	1.26 (1.15–1.38)	<0.001
Homonymous hemianopsia	1.43 (1.33–1.54) *	<0.001
Deep vein thrombosis	3.97 (3.76–4.20) *	<0.001
Urinary tract infection	0.89 (0.85–0.94)	<0.001
Pneumonia	0.84 (0.78–0.90)	<0.001
Palliative care	0.57 (0.53–0.61)	<0.001
Intubation	0.68 (0.63–0.74)	<0.001
Tracheostomy	0.70 (0.59–0.84)	<0.001

Legend: * most robust independent predictors increasing the likelihood of PFO presence.

## Data Availability

The data used in this analysis may be purchased from the Healthcare Cost and Utilization Project (HCUP) website. The authors do not have permission to share the data used for the analysis.

## Appendix A

**Table A1:** Codes for data analysis and their sources.

Variable	Source	ICD-10 Code
Patent foramen ovale	I10_DX1/40	Q21.1
Acute cerebral infarction	I10_DX1/40	I63*
Age	NIS Core	AGE
Female sex	NIS Core	FEMALE
Race	NIS Core	RACE
Elective admission	NIS Core	ELECTIVE
Weekend admission	NIS Core	AWEEKEND
Season of admission	NIS Core	AMONTH where Spring was Mar-May, Summer was June-Aug, Fall was Sept-Nov, Winter was Dec-Feb.
Primary expected payer	NIS Core	PAY1
ZIP income quartile	NIS Core	ZIPINC_QRTL
Hospital region	NIS Hospital	HOSP_REGION
Hospital bed size	NIS Hospital	HOSP_BEDSIZE
Rural	NIS Core	PL_NCHS
Teaching hospital	NIS Hospital	HOSP_LOCTEACH
Nicotine dependence	I10_DX1/40	Z72.0
Alcohol misuse	I10_DX1/40	F10.1
Obesity	I10_DX1/40	E66.0, E66.1, E66.2, E66.8, E66.9
Hypertension	I10_DX1/40	I10*, I11*, I12*, I13*, I15*, I16*
Hyperlipidemia	I10_DX1/40	E78.0*, E78.1, E78.2, E78.3, E78.4*, E78.5
Diabetes mellitus	I10_DX1/40	E08*, E09*, E10*, E11*, E13*
Previous myocardial infarction	I10_DX1/40	I25.2
Previous heart failure	I10_DX1/40	I09.81, I11.0, I50*
Atrial fibrillation or flutter	I10_DX1/40	I48*
Previous stroke	I10_DX1/40	Z86.73, I69*
Atherosclerosis of the aorta	I10_DX1/40	I70.0
Peripheral vascular disease	I10_DX1/40	I73*
Chronic kidney disease	I10_DX1/40	N18*
Liver failure	I10_DX1/40	K72*
Chronic lung disease	I10_DX1/40	J40*–J47*
Any cancer	I10_DX1/40	C00*–C96*
Dementia	I10_DX1/40	F01*, F02*, F03*, G30*, G31*
Aphasia	I10_DX1/40	I69.320, I69.920, R47.01
Hemiplegia	I10_DX1/40	I69.35, G81*
Neglect	I10_DX1/40	R41.4
Stupor	I10_DX1/40	R40*
Dysphagia	I10_DX1/40	R13.1
Homonymous hemianopsia	I10_DX1/40	H53.46
Deep vein thrombosis	I10_DX1/40	I82*
Urinary tract infection	I10_DX1/40	N30.0, N30.9, N34.1, N34.2, N39.0
Pneumonia	I10_DX1/40	J12*–J18*
Palliative care	I10_DX1/40	Z51.5
Intubation	I10_DX1/40I10_PR1/25	Z99.110BH17EZ
Tracheostomy	I10_PR1/25	0B110F4
Hospital transfer	NIS Core	TRAN_IN
Thrombolysis	I10_PR1/25	3E03317, 3E04317
Mechanical thrombectomy	I10_PR1/25	03CG3ZZ, 03CG3Z7, 03CG4ZZ, 03CH3Z7, 03CJ0ZZ, 03CJ3ZZ, 03CK3Z7, 03CK3ZZ, 03CL3Z7, 03CL3ZZ, 03CL0ZZ, 03CP3ZZ, 03CY3ZZ, 00C73ZZ
PFO closure	I10_PR1/25	02Q50, 02Q53, 02Q54
Intracranial hemorrhage	NIS Core	I60*, I61*, I62.9
Discharge home self-care	NIS Core	DISPUNIFORM = home or self-care and not palliative
In-hospital mortality	NIS Core	DIED

* Asterisk denotes that all subclassifications of the parent code were included.
